# ESKAPEE pathogens newly released from biofilm residence by a targeted monoclonal are sensitized to killing by traditional antibiotics

**DOI:** 10.3389/fmicb.2023.1202215

**Published:** 2023-07-26

**Authors:** Nikola Kurbatfinski, Cameron N. Kramer, Steven D. Goodman, Lauren O. Bakaletz

**Affiliations:** ^1^Center for Microbial Pathogenesis, Abigail Wexner Research Institute at Nationwide Children's Hospital, Columbus, OH, United States; ^2^Department of Pediatrics, The Ohio State University College of Medicine, Columbus, OH, United States

**Keywords:** AMR, MRSA, humanized monoclonal antibody, DNABII proteins, tip-chimer peptide

## Abstract

**Introduction:**

The “silent” antimicrobial resistance (AMR) pandemic is responsible for nearly five million deaths annually, with a group of seven biofilm-forming pathogens, known as the ESKAPEE pathogens, responsible for 70% of these fatalities. Biofilm-resident bacteria, as they exist within the disease site, are canonically highly resistant to antibiotics. One strategy to counter AMR and improve disease resolution involves developing methods to disrupt biofilms. These methods aim to release bacteria from the protective biofilm matrix to facilitate their killing by antibiotics or immune effectors. Several laboratories working on such strategies have demonstrated that bacteria newly released from a biofilm display a transient phenotype of significantly increased susceptibility to antibiotics. Similarly, we developed an antibody-based approach for biofilm disruption directed against the two-membered DNABII family of bacterial DNA-binding proteins, which serve as linchpins to stabilize the biofilm matrix. The incubation of biofilms with α-DNABII antibodies rapidly collapses them to induce a population of newly released bacteria (NRel).

**Methods:**

In this study, we used a humanized monoclonal antibody (HuTipMab) directed against protective epitopes of a DNABII protein to determine if we could disrupt biofilms formed by the high-priority ESKAPEE pathogens as visualized by confocal laser scanning microscopy (CLSM) and COMSTAT2 analysis. Then, we demonstrated the potentiated killing of the induced NRel by seven diverse classes of traditional antibiotics by comparative plate count.

**Results:**

To this end, ESKAPEE biofilms were disrupted by 50%−79% using a single tested dose and treatment period with HuTipMab. The NRel of each biofilm were significantly more sensitive to killing than their planktonically grown counterparts (heretofore, considered to be the most sensitive to antibiotic-mediated killing), even when tested at a fraction of the MIC (1/250–1/2 MIC). Moreover, the bacteria that remained within the biofilms of two representative ESKAPEE pathogens after HuTipMab disruption were also significantly more susceptible to killing by antibiotics.

**Discussion:**

New data presented in this study support our continued development of a combinatorial therapy wherein HuTipMab is delivered to a patient with recalcitrant disease due to an ESKAPEE pathogen to disrupt a pathogenic biofilm, along with a co-delivered dose of an antibiotic whose ability to rapidly kill the induced NRel has been demonstrated. This novel regimen could provide a more successful clinical outcome to those with chronic, recurrent, or recalcitrant diseases, while limiting further contribution to AMR.

## 1. Introduction

Bacterial antimicrobial resistance (AMR) is a global concern of such magnitude that it has been referred to as a confoundingly “silent” pandemic due to the relatively limited attention that it has received (Semedo and Bury, 2021; UK Health Security Agency, 2021; International Federation of Pharmaceutical Manufacturers Associations, 2022; Laxminarayan, 2022). A systematic analysis of the worldwide burden of AMR conducted in 2019 (Antimicrobial Resistance Collaborators, 2022) estimated that 4.95 million deaths were *associated* with AMR (i.e., the number of deaths if all drug-resistant infections are replaced by no infection) and 1.27 million deaths were directly *attributable* to it (i.e., the number of deaths if all drug-resistant infections are replaced by drug-susceptible infections).

Multifaceted causes of increasing AMR can be attributed to several factors, including societal factors (industrialization and population density), genetic factors (mutations, horizontal gene transfer (HGT), and selective pressure), ecological factors (antibiotic use in agriculture and improper disposal of antibiotics), and overuse and misuse of antibiotics (access without prescription, use after prescribed treatment window, and over-prescription). A major contributor to AMR stems from our efforts to treat diseases, particularly, in cases where biofilms play a significant role in pathogenesis, recurrence, and chronicity, with antibiotics as the only option as no other options are currently available (Barbosa and Levy, 2000; Michael et al., 2014; Uruen et al., 2020). While the causative bacterial agent is typically highly sensitive when grown planktonically in a rich medium, as tested in a clinical microbiology lab, at the disease site, bacteria commonly reside within a biofilm, which contributes significantly to resistance to the recommended antibiotic treatment (Macia et al., 2014; Ribeiro da Cunha et al., 2019; WHO, 2019).

Biofilms are three-dimensional communities of bacteria formed on surfaces (biotic or abiotic); they may also exist in an untethered aggregate state [e.g., biofilms resident in the lung and/or sputum (Kolpen et al., 2022)]. Biofilm-resident bacteria are encased within a self-produced extracellular polymeric substance (EPS) consisting of exopolysaccharides, proteins, lipids, and, commonly, extracellular DNA (eDNA), among others (Whitchurch et al., 2002; Flemming and Wingender, 2010; Boisvert et al., 2016; Gunn et al., 2016). This preferred bacterial lifestyle promotes diverse mechanisms of tolerance to both antibiotics and immune effectors, which include maintaining a quiescent metabolism, using quorum sensing, and producing an EPS that limits the access of antibiotics and immune effectors (Stewart, 2002; Davies, 2003; Rutherford and Bassler, 2012; Jamal et al., 2018; Orazi and O'Toole, 2019; Sharma et al., 2019; Uruen et al., 2020). Moreover, the targets of specific antibiotics, such as the cell wall and/or protein synthesis, are often not actively expressed by bacteria in a quiescent metabolic state (Eng et al., 1991). Similarly, quorum sensing can result in the upregulation of genes whose products contribute to resistance, such as the efflux pumps that move antibiotics out of the cell (Uruen et al., 2020; Zhao et al., 2020). Thus, a >1,000-fold increase in antibiotic concentration is required to kill biofilm-resident bacteria compared to their planktonic, or free-living, counterparts (Nickel et al., 1985; Ceri et al., 1999; Moskowitz et al., 2004; Hoiby et al., 2010; Hengzhuang et al., 2011, 2012).

The recalcitrance of bacteria within biofilms to antibiotics and immune effectors is of tremendous concern as up to 80% of human bacterial infections have a biofilm component (Costerton et al., 1999; Dongari-Bagtzoglou, 2008). Furthermore, biofilm recalcitrance promotes the long-term survival of bacteria on abiotic surfaces, including medical devices, as found in clinical settings; this can serve as a nidus for HGT and contribute to the continued spread of AMR (Babapour et al., 2016; Alcantar-Curiel et al., 2018). Despite extensive efforts, no strategies or agents have been proven completely successful in effectively treating, or ideally preventing, recalcitrant biofilm diseases. As such, treatment with traditional antibiotics is still the standard of care, despite widespread failed clinical efficacy (Macia et al., 2014; Ribeiro da Cunha et al., 2019; WHO, 2019). One potential strategy to overcome these obstacles is the development of a broadly effective, and ideally, species-agnostic methodology to disrupt biofilms and release the resident bacteria so that they can be killed by traditional antibiotics that were ineffective when these pathogens resided within a biofilm.

This overall approach is promising as several laboratories have shown that bacteria newly released from a biofilm, regardless of the specific mode of release, demonstrate a transient phenotype of significantly increased susceptibility to antibiotics (Marks et al., 2013; Zemke et al., 2014, 2020; Chambers et al., 2017; Fleming et al., 2017; Howlin et al., 2017; Fleming and Rumbaugh, 2018; Goodwine et al., 2019; Redman et al., 2021). Similarly, we demonstrated that, if we disrupt a biofilm formed by minimally passaged non-typeable *Haemophilus influenzae* (NTHI), a predominant respiratory tract pathogen (Pichichero et al., 2008; Frost et al., 2022), using an antibody targeting a ubiquitous structural protein of the biofilm matrix, the newly released (or NRel) bacteria (Mokrzan et al., 2020) NTHI exhibits significant susceptibility to *in vitro* killing by trimethoprim/sulfamethoxazole, amoxicillin, ampicillin, or cefdinir (Goodman et al., 2011; Brockson et al., 2014; Mokrzan et al., 2020). These antibiotics were unable to kill the NTHI isolate when it resided within a biofilm (Mokrzan et al., 2020).

The antibody we used for biofilm disruption targets an essential structural component of biofilm (Goodman et al., 2011; Brockson et al., 2014; Novotny et al., 2019a,b), the two bacterial DNA-binding proteins known as the DNABII family. Extracellularly, the DNABII proteins [histone-like protein (HU) and integration host factor (IHF)] bind to and bend double-stranded DNAs (Swinger and Rice, 2004; Dey et al., 2017; Devaraj et al., 2018) within the biofilm EPS; thus, they provide essential support to the biofilm matrix (Whitchurch et al., 2002; Flemming and Wingender, 2010; Gunn et al., 2016; Devaraj et al., 2018). The incubation of biofilms with DNABII-targeted antibodies does not kill resident bacteria (Goodman et al., 2011; Novotny et al., 2013; Brockson et al., 2014); instead, it induces an equilibrium shift of DNABII proteins from their eDNA-bound state in the biofilm matrix to an unbound state in the extracellular milieu, resulting in rapid biofilm collapse and NRel release. To date, we have demonstrated, based on the plate count and image analysis, that the use of this antibody at a single dose and incubation time effectively disrupts biofilms formed by 23 bacterial genera *in vitro* between 57 and 91% (Goodman et al., 2011; Gustave et al., 2013; Novotny et al., 2013; Brockson et al., 2014; Devaraj et al., 2015, 2021; Freire et al., 2017; Rocco et al., 2018; Kurbatfinski et al., 2022). We have also demonstrated the effective clearance of both bacteria and the biofilm matrix *in vivo* by using three distinct pre-clinical models of human disease wherein no supplemental antibiotics were used (Goodman et al., 2011; Novotny et al., 2016, 2019a, 2020, 2021; Freire et al., 2017).

In a recent study, we demonstrated that NRel induced by the incubation of biofilms formed by six common respiratory tract pathogens with a DNABII-targeted humanized monoclonal antibody were sensitized to killing by antibiotics (Kurbatfinski et al., 2022). In this study, we focus our efforts on the seven highly virulent, biofilm-forming ESKAPEE pathogens (*Enterococcus faecium, Staphylococcus aureus, Klebsiella pneumoniae, Acinetobacter baumannii, Pseudomonas aeruginosa, Enterobacter* spp., and *Escherichia coli*), which are often multi-drug resistant (MDR) and thereby typically “escape” antibiotic treatment (Rice, 2008; Boucher et al., 2009; Marturano and Lowery, 2019). The WHO considers ESKAPEE pathogens “critical” and “high” priorities against which the development of new therapeutic strategies must be targeted (Tacconelli et al., 2018). These pathogens are responsible for the majority of healthcare-associated infections (HCAIs) (Pendleton et al., 2013; Haque et al., 2018) and are particularly concerning when indwelling medical devices are implanted (Pendleton et al., 2013). In 2011, ESKAPEE pathogens were responsible for nearly half of all HCAIs in the United States (Magill et al., 2014), and in 2019, they caused nearly 70% of deaths associated with or attributable to AMR, as well as ~10% of all global deaths (Antimicrobial Resistance Collaborators, 2022; GBD Antimicrobial Resistance Collaborators, 2022). In recent publications, depending on the age of the biofilm and the relative concentration and/or duration of treatment with anti-DNABII antisera, we have demonstrated that biofilms formed by each of the ESKAPEE pathogens could be disrupted by 37%−100% (Goodman et al., 2011; Novotny et al., 2016, 2020; Devaraj et al., 2019, 2021; Kurbatfinski et al., 2022). In this study, we investigated whether the treatment of ESKAPEE biofilms with the humanized monoclonal antibody that targets protective epitopes of a DNABII protein (e.g., “HuTipMab”) can induce the formation of NRel that are similarly highly vulnerable to antibiotic-mediated killing.

## 2. Materials and methods

### 2.1. Antibodies

A humanized monoclonal antibody (HuTipMab) of the IgG isotype against a tip-chimer peptide, which was designed to mimic the protective epitopes within the DNA-binding tips of the alpha and beta subunits of a bacterial DNABII family member, was engineered from a similarly directed murine monoclonal antibody (Novotny et al., 2020). It was then produced for us by Lake Pharma, Inc. (San Carlos, CA).

### 2.2. Antibiotics

Amikacin sulfate salt (AMK, Matrix Scientific, Columbia, SC), ceftazidime hydrate (CAZ, LKT Laboratories Inc., St Paul, MN), colistin sulfate salt (CST, Sigma-Aldrich, St. Louis, MO), imipenem monohydrate (IPM, LKT Laboratories Inc., St Paul, MN), piperacillin sodium salt (PIP, Alfa Aesar, Haverhill, MA), tobramycin (TOB, MilliporeSigma, Burlington, MA), and vancomycin hydrochloride (VAN, Alfa Aesar, Haverhill, MA) were stored according to the manufacturer's instructions; they were suspended and diluted in Mueller-Hinton II Broth (cation-adjusted; BD BBL™, Franklin Lakes, NJ) immediately before use. Levofloxacin (TCI America, Inc., Portland, OR), linezolid (LZD, ACROS Organics, Fairlawn, NJ), trimethoprim (Sigma-Aldrich, St. Louis, MO), and sulfamethoxazole (Santa Cruz Biotech, Dallas, TX) were stored according to the manufacturer's instructions, suspended in dimethyl sulfoxide (Fisher Scientific International, Inc., Hampton, NH), and then further diluted 1:1,000 in cation-adjusted Mueller-Hinton II Broth (CAMHB) immediately before use. Trimethoprim/sulfamethoxazole (SXT) was prepared in a 1:19 ratio in accordance with EUCAST guidelines (ISO, 2019; EUCAST, 2020). The MIC value for trimethoprim/sulfamethoxazole is expressed as the concentration (μg/ml) of trimethoprim. See Table 1.

**Table 1:** Minimal inhibitory concentration values for indicated antibiotics against planktonically grown ESKAPEE pathogens.

	**Antibiotics (**μ**g/ml)**									
**QC organism/strain**	**LVX**	**CAZ**	**VAN**	**IPM**	**TOB**	**LZD**	**PIP**	**AMK**	**SXT**	**CST**
*Pseudomonas aeruginosa* 27853^a^	32	8	NA^b^	16	4	4,096	16	8	16	1
*Staphylococcus aureus* 29213^a^	0.5	64	1	64	2	4	8	32	0.25	NA^b^
	**Antibiotics (**μ**g/ml)**									
**ESKAPEE pathogens**										
*Klebsiella pneumoniae*	0.25	4	…	…	…	…	…	4	…	…
*Staphylococcus aureus* (MRSA)	0.125	…	1	…	…	2	128	…	…	…
*Enterobacter* sp.	4	…	…	4,096	2	…	…	…	…	…
*Escherichia coli*	0.0625	1	…	…	…	…	…	…	0.125	…
*Acinetobacter baumannii*	0.25	…	…	…	1	…	…	…	…	1
*Enterococcus faecium*	…	2,048	0.5	16	…	…	…	…	…	…
*Pseudomonas aeruginosa*	…	2	…	…	2,048	…	2	…	…	…

AMK, amikacin; CAZ, ceftazidime; CST, colistin; colistin; IPM, imipenem; LVX, levofloxacin; LZD, linezolid; PIP, piperacillin; TOB, tobramycin; SXT, trimethoprim/sulfamethoxazole; VAN, vancomycin.

^a^ATCC strains used as quality control standards in accordance with EUCAST guidelines (ISO, 2019; EUCAST, 2020).

^b^not applicable as CST is specific for gram-negative bacteria and VAN is specific for gram-positive bacteria.

… indicates organism and antibiotic combination not assayed.

### 2.3. Bacterial strains and sources

See Supplementary Table 1.

### 2.4. Preparation of biofilms *in vitro*

All bacteria were grown on either tryptic soy agar (TSA; MRSA*, P. aeruginosa, Enterobacter* sp.), Lysogeny broth (LB) agar [*A. baumannii, E. coli* (Goodman et al., 2011)], or brain heart infusion agar (*E. faecium, K. pneumoniae*) for 18–24 h at 37°C, 5% CO_2_, in a humidified atmosphere. Well-isolated colonies were picked and then inoculated into their respective liquid media (tryptic soy broth for MRSA, *P. aeruginosa*, and *Enterobacter* sp.; Lysogeny broth for *A. baumannii* and *E. coli*; and brain heart infusion broth for *E. faecium* and *K. pneumoniae*). Bacteria were suspended by pipetting up and down and then subjected to gentle sonication in a water bath sonicator (ultrasonic bath 2.8 L, Fisher Scientific) for 2 min to disassociate any aggregates. After sonication, OD_490_ was adjusted to 0.1, and then, the bacteria were further diluted in their respective liquid media to a final concentration of 2 × 10^5^ CFU/ml, as confirmed by plate count. A total of 200 μl of this suspension was added to each well of a 48-well microtiter plate or 8-well chambered cover-glass slide (Cellvis, Mountain View, CA) and then incubated statically for 16 h at 37°C and 5% CO_2_ in a humidified atmosphere.

### 2.5. Determination of MIC

MIC values were determined as described in a previous study (Kurbatfinski et al., 2022), in accordance with the EUCAST guidelines (ISO, 2019; EUCAST, 2020). Briefly, increasing two-fold dilutions of antibiotic solutions were placed into an untreated, round bottom, 96-well plate (Corning, Corning, NY). Each bacterial species was diluted to ~1 × 10^6^ CFU/ml in CAMHB, and an equal volume (50 μl) of the inoculum was placed into the pre-filled wells within 30 min of inoculum preparation. After 20 h of incubation (37°C, 5% CO_2_, humidified), the plates were viewed under a direct light source, and the lowest concentration of antibiotic that prevented visible growth was recorded as the MIC. Final MIC values were expressed in μg/ml based on the calculation of the geometric mean of three biological replicates.

This assay was performed for each of the 10 antibiotics selected against a well-characterized isolate of both *P. aeruginosa* (strain 27853) and *S. aureus* (strain 29213) used here as quality control standards, again in accordance with EUCAST guidelines (EUCAST, 2020) (Table 1).

### 2.6. Determination of potentially increased sensitivity of NRel and residual biofilm to antibiotic-mediated killing

To allow us to readily detect the increased killing of NRel bacteria by each antibiotic tested, we determined a concentration of antibiotic that would limit the killing of each planktonically grown bacterial species to no >~35%. Briefly, 50 μl of planktonically grown bacteria prepared as described above for the MIC assay was placed into a 96-well plate that contained an equal volume of CAMHB alone (growth control) or CAMHB supplemented with antibiotic at a fraction of the MIC. After 2-h incubation (37°C, 5% CO_2_, humidified), 96-well plates were sonicated for 2 m in a water bath sonicator, and then they were diluted and plated. This assay was repeated with different fractions of the MIC until the planktonically grown bacteria were at or below the 35% killing threshold.

To determine the susceptibility of HuTipMab ESKAPEE NRel to antibiotic-mediated killing, we first needed to collect NRel. To do so, the medium was removed from above 16-h biofilms formed within a 48-well microtiter plate, and the biofilms were gently washed twice with 200 μl equilibrated 1X Dulbecco's phosphate-buffered saline (DPBS) without calcium or magnesium (Corning, Corning, NY). This was performed to remove non-biofilm-resident bacteria before adding 5 μg HuTipMab per 0.8 cm^2^ well in 200 μl CAMHB. After 30-min of incubation, the supernatant that contained the NRel was carefully collected, gently sonicated in a water bath sonicator for 5 min to disrupt aggregates, and then diluted to 1 × 10^6^ CFU/ml. The assay described above for planktonic populations was repeated at the pre-determined antibiotic concentration with the planktonic and HuTipMab NRel populations. Percent killing was calculated as follows: CFU/ml growth control medium – CFU/ml of samples incubated with antibiotic divided by CFU/ml growth control medium × 100. Percent killing is reported as the mean of three biological replicates with three technical replicates each ± SEM.

To determine the relative sensitivity to antibiotic-mediated killing of bacteria resident within the biofilms formed by MRSA or *P. aeruginosa* after disruption by HuTipMab, 16-h biofilms formed by either pathogen in an 8-well chambered cover-glass slide were gently washed twice, as described above. Then, the medium alone or 5 μg of HuTipMab in the medium was added to the wells and incubated statically at 37°C and 5% CO_2_ in a humidified atmosphere for 30 min. Subsequently, the supernatant was removed, and the remaining biofilm was gently washed twice. Next, 200 μl of antibiotics at the tested doses or the medium alone (growth control) were added. After 2-h incubation at 37°C and 5% CO_2_ in a humidified atmosphere, cover glass slides were removed, and each well was scraped along every surface using a 200-μl pipet tip on a pipettor while repeatedly aspirating and releasing a 100-μl volume of the medium, either alone, or with antibiotics, to dissociate any adherent bacteria. Following dissociation, the entire cover glass slide was degassed for 5 min to remove any resulting dissolved gases and sonicated for 5 min to disrupt aggregates; then, the samples were diluted and plated (Kragh et al., 2019). Percent killing was calculated as follows: CFU/ml growth control medium minus CFU/ml of samples incubated with antibiotic divided by CFU/ml growth control medium × 100. Percent killing is reported as the mean of three biological replicates with two technical replicates each ± the SEM.

### 2.7. Confocal laser scanning microscopy and the analysis of relative biofilm disruption

The procedures described previously were followed to wash 16-h biofilms, which were incubated with either the medium alone or 5 μg HuTipMab/well for 30 min and then stained with the bacterial membrane stain FM 1-43FX (green). The stain was removed after 15 min, the wells were washed twice with DPBS, and the stained biofilms were fixed for 2 h (1.6% paraformaldehyde, 2.5% glutaraldehyde, and 4% acetic acid in 0.1 M phosphate buffer) (Brockson et al., 2014; Novotny et al., 2019b; Kurbatfinski et al., 2022). After 2 h, the fixative was removed, DPBS was added, and the biofilms were visualized by confocal laser scanning microscopy (CLSM) with a ZEISS CLSM800 microscope. Biomass values (μm^3^/μm^2^) were calculated using COMSTAT2, and the mean percent biofilm disruption was calculated as [(the biomass of wells incubated with the medium alone – the biomass of wells incubated with HuTipMab) divided by the biomass of wells incubated with the medium alone] × 100. Mean biofilm disruption is reported as the mean of three biological replicates with two technical replicates each ±SEM.

### 2.8. Statistical analyses

All statistical analyses were performed with GraphPad (Prism) software V9. The MIC values were calculated based on the geometric mean of at least three biological replicates. Comparisons of relative percent killing between planktonic and NRel were determined by conducting Welch's *t*-test or two-way ANOVA.

## 3. Results

### 3.1. Determination of MIC values for each of the 10 selected antibiotics

To ascertain whether ESKAPEE NRel were more sensitive to antibiotic-mediated killing than their planktonically grown counterparts, we first established the relative minimal inhibitory concentration (MIC) value (for each of the 10 antibiotics of seven classes selected for use) via the assay performed in clinical microbiology laboratories to guide antibiotic selection for clinical use. To do so, we followed EUCAST protocols (Wiegand et al., 2008; ISO, 2019) and used reference strains *P. aeruginosa* 27853 and *S. aureus* 29213 as quality controls (Table 1). We determined the relative inhibition of growth by each of the three antibiotics typically chosen for patients with disease due to each ESKAPEE pathogen. We then used these MIC values to identify the concentration that would restrict the killing of planktonically grown ESKAPEE pathogens to ≤35% to facilitate the detection of the increased killing of NRel.

### 3.2. Potentiated killing of ESKAPEE NRel by antibiotics

**Figure 1:**
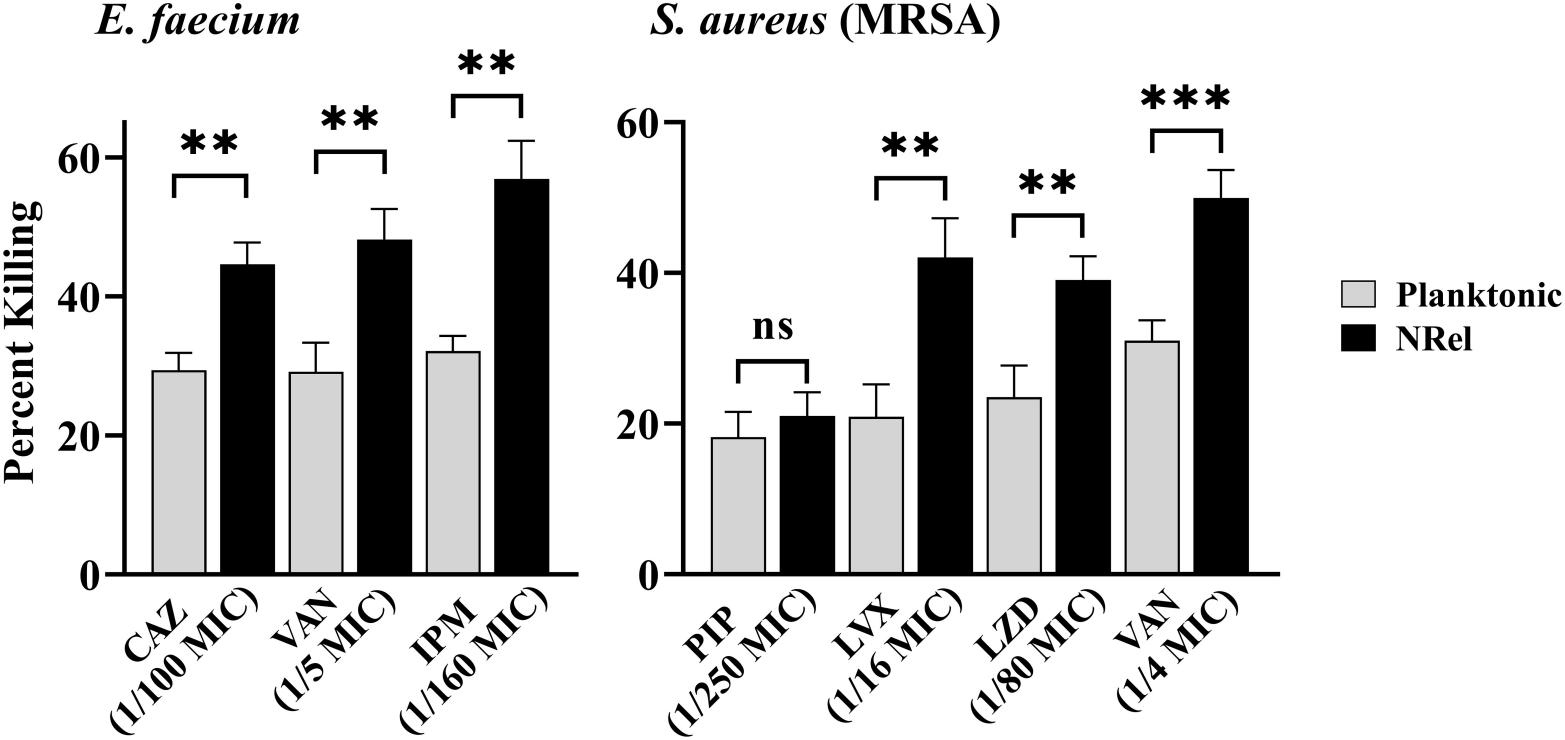
Relative percent killing of HuTipMab-induced *E. faecium* and MRSA NRel. NRel released from biofilms formed by *E. faecium* or MRSA by the action of HuTipMab were significantly more sensitive to killing by all antibiotics tested than their isogenic planktonically grown counterparts. In this assay, we also tested MRSA NRel for relative killing by piperacillin (PIP) to confirm its innate resistance to a β-lactam antibiotic and as a negative control, since this resistance is genetically encoded and not affected by release from biofilm residence. As expected, there was no significant difference in the relative killing of MRSA by PIP between the planktonically grown state and the NRel state. Antibiotics were used at a fraction of the planktonic MIC as indicated on the *x*-axis; the final assay concentrations for both NRel and planktonic populations tested were ~5 × 10^5^ CFU/ml. Statistically significant differences in percent killing are reported as **P ≤ 0.01 and ***P ≤ 0.005.

To determine if ESKAPEE HuTipMab-induced NRel were killed more readily by antibiotics than their planktonically grown counterparts, both populations were incubated with the concentration that limited the killing of the corresponding planktonic population to ≤35% in a 2-h assay. When this single dose and treatment period were used, ESKAPEE biofilms were disrupted by 50%−79% (Supplementary Table 2), as determined by CSLM analysis using COMSTAT2. When HuTipMab-induced *E. faecium* NRel were tested with three antibiotics that are currently used clinically for *E. faecium* infections [e.g., urinary tract infection, bacteremia, and endocarditis (Agudelo Higuita and Huycke, 2014)], they were significantly more sensitive to each antibiotic than their planktonically grown counterparts. The killing of the planktonically grown *E. faecium* by any tested antibiotic did not exceed 32%. However, the killing of *E. faecium* NRel by ceftazidime (CAZ), a third-generation cephalosporin that targets cell wall synthesis (Hutchings et al., 2019), was significantly greater at 45% (*P* ≤ 0.01). In comparison, vancomycin (VAN), a cell wall synthesis-targeted antibiotic, and imipenem (IPM), a carbapenem antibiotic, achieved a killing rate of 48 and 57%, respectively (*P* ≤ 0.01; Figure 1, left panel). Note that this significantly increased susceptibility to killing by CAZ, VAN, and IPM was achieved when they were used at 1/100, 1/5, and 1/160 of the planktonically determined MIC, respectively.

Next, we tested a methicillin-resistant isolate of *S. aureus* (MRSA) after confirming its resistance to the β-lactam antibiotic piperacillin (PIP). The planktonic MIC was 128 μg PIP/ml (any value >16 is considered resistant) (Muller et al., 2015). As expected, HuTipMab-induced MRSA NRel maintained this genetically conferred resistance to PIP. The release from biofilm residence did not affect this form of antibiotic resistance and thus served as a negative control in this study (Figure 1, right panel). HuTipMab-induced MRSA NRel were significantly more sensitive to killing by the quinolone levofloxacin (LVX), which targets DNA gyrase and topoisomerase IV to prevent DNA replication (Hutchings et al., 2019), the oxazolidinone antibiotic linezolid (LZD), which inhibits protein synthesis by translation disruption and also by VAN. The relative percent killing of MRSA NRel by LVX, LZD, and VAN was 42%, 39%, and 50%, respectively, compared to 21%, 24%, and 31% for MRSA grown planktonically (*P* ≤ 0.01, 0.001, and 0.005, respectively). All antibiotics were tested at a fraction of the planktonically determined MIC (e.g., 1/16 for LVX, 1/80 for LZD, and 1/4 for VAN).

**Figure 2:**
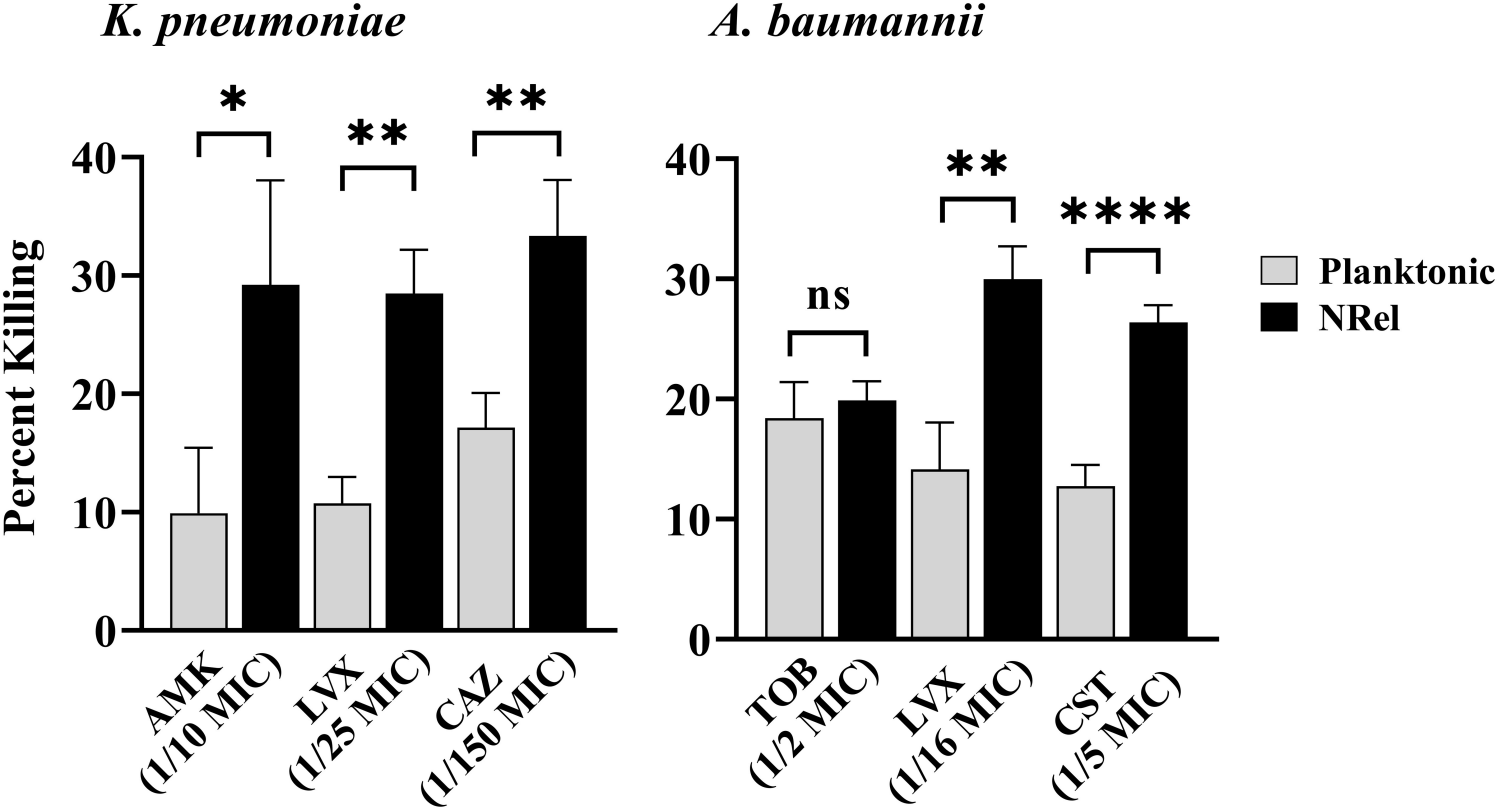
Relative percent killing of HuTipMab-induced *K. pneumonia* and *A. baumannii* NRel. When tested with three antibiotics of different classes, the relative percent killing of HuTipMab-induced NRel from biofilms formed by *K. pneumoniae* or *A. baumannii* was significantly higher than that of their planktonically grown counterparts, except for the killing of *A. baumannii* NRel by TOB wherein the killing was equivalent. Antibiotics were used at a fraction of the planktonic MIC as indicated on the *x*-axis; the final assay concentrations for both NRel and planktonic populations tested were 5 × 10^5^ CFU/ml. Statistically significant differences in percent killing are reported as *P ≤ 0.05; **P ≤ 0.01; and ****P ≤ 0.001.

Similarly, HuTipMab induced the NRel of a *K. pneumoniae* isolate, which displayed heightened susceptibility to killing by amikacin (AMK), an aminoglycoside that irreversibly binds to the 30S subunit to prevent protein synthesis (Hutchings et al., 2019). Furthermore, NRel displayed heightened susceptibility to LVX and CAZ (Figure 2, left panel). The relative percent killing of *K. pneumoniae* NRel was 29%, 29%, and 33% by AMK, LVX, and CAZ, respectively, compared to 10%, 11%, and 17%, respectively, in the planktonically grown condition (*P* ≤ 0.05, 0.01, and 0.01, respectively). All antibiotics were tested at a fraction of the planktonically determined MIC (e.g., 1/10 for AMK, 1/25 for LVX, and 1/150 for CAZ).

HuTipMab-induced NRel of a clinical isolate of *A. baumannii* with tobramycin (TOB), LVX, and colistin (CST) also demonstrated the vulnerable NRel phenotype (Figure 2, right panel). When tested with the aminoglycoside, TOB, which prevents protein synthesis (Hutchings et al., 2019), *A. baumannii* were rendered as sensitive when planktonically grown with the killing of planktonic *A. baumannii* at 18% and NRel at 20%. However, the killing of *A. baumannii* NRel by LVX and CST, a polycationic peptide that solubilizes the cytoplasmic membrane (Hutchings et al., 2019), was 30% and 26%, respectively, whereas that for planktonically grown *A. baumannii* was 14% and 13%, respectively (*P* ≤ 0.01 and *P* ≤ 0.001, respectively). All antibiotics were used at fractions of the planktonically determined MIC: 1/2 MIC for TOB, 1/16 MIC for LVX, and 1/5 MIC for CST.

**Figure 3:**
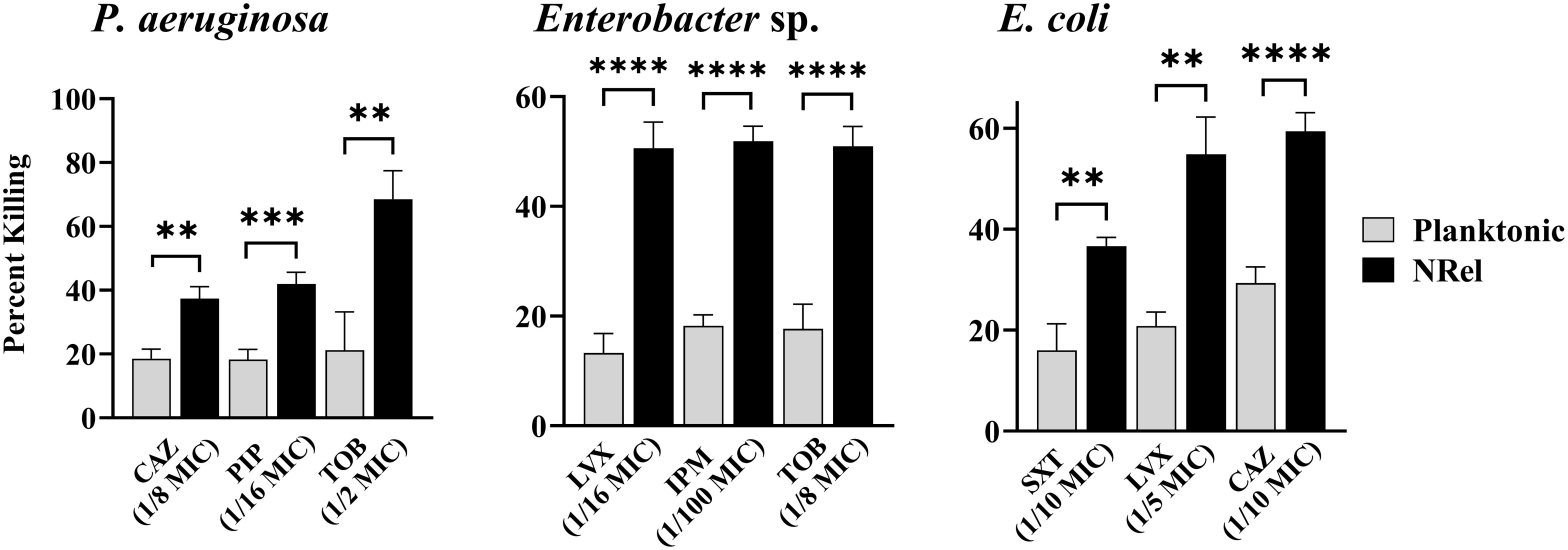
Relative percent killing of HuTipMab-induced *P. aeruginosa, Enterobacte* sp., and *E. coli* NRel. HuTipMab-induced NRel from biofilms formed by *P. aeruginosa, Enterobacter* sp., and *E. coli* also expressed a phenotype of significant sensitivity to killing by each of the three tested antibiotics compared to their isogenic planktonically grown counterparts. Antibiotics were used at a fraction of the planktonic MIC as indicated on the *x*-axis; the final assay concentrations for both NRel and planktonic populations tested were 5 × 10^5^ CFU/ml. Statistically significant differences in percent killing are reported as **P ≤ 0.01; ***P ≤ 0.005; and ****P ≤ 0.001.

Finally, we assessed the relative antibiotic sensitivity of HuTipMab-induced NRel of a highly TOB-resistant clinical isolate of *P. aeruginosa* (MIC of 2048 μg/ml) and a clinical isolate of both *Enterobacter* sp. and *E. coli* (Figure 3). *Pseudomonas aeruginosa* NRel were significantly more sensitive to CAZ, PIP, and TOB with relative percent killing values of 37%, 42%, and 69%, respectively, compared to the planktonically grown counterparts, which had values of 19%, 18%, and 21%, respectively (*P* ≤ 0.01, 0.005, and 0.01, respectively; Figure 3, left panel). CAS, PIP, and TOB were used at 1/8, 1/16, and 1/2 the planktonic MIC, respectively.

*Enterobacter* sp. and *E. coli* NRel followed similar trends. *Enterobacter* sp. NRel were significantly more sensitive to killing by LVX, IPM, and TOB than their planktonically grown counterparts (*P* ≤ 0.001). The killing of planktonic *Enterobacter* sp. was 13%, 18%, and 18%, respectively, whereas the killing of *Enterobacter* sp. NRel was 51%, 52%, and 51%, respectively, when tested with LVX at 1/16 MIC, IPM at 1/100 MIC, and TOB at 1/8 MIC (Figure 3, middle panel). For *E. coli*, we tested trimethoprim/sulfamethoxazole (SXT; a synergistic combination of two sulfonamides that targets the successive steps of the folate synthesis pathway) (Hutchings et al., 2019), LVX, and CAZ. HuTipMab-induced *E. coli* NRel were significantly more sensitive to killing than their isogenic planktonically grown counterparts as the relative killing of *E. coli* NRel by SXT, LVX, and CAZ was 37%, 55%, and 59%, respectively, whereas that of planktonic *E. coli* was 16%, 21%, and 29%, respectively (*P* ≤ 0.01 to 0.001). SXT was tested at 1/10 MIC, LVX at 1/5 MIC, and CAZ at 1/10 MIC.

**Figure 4:**
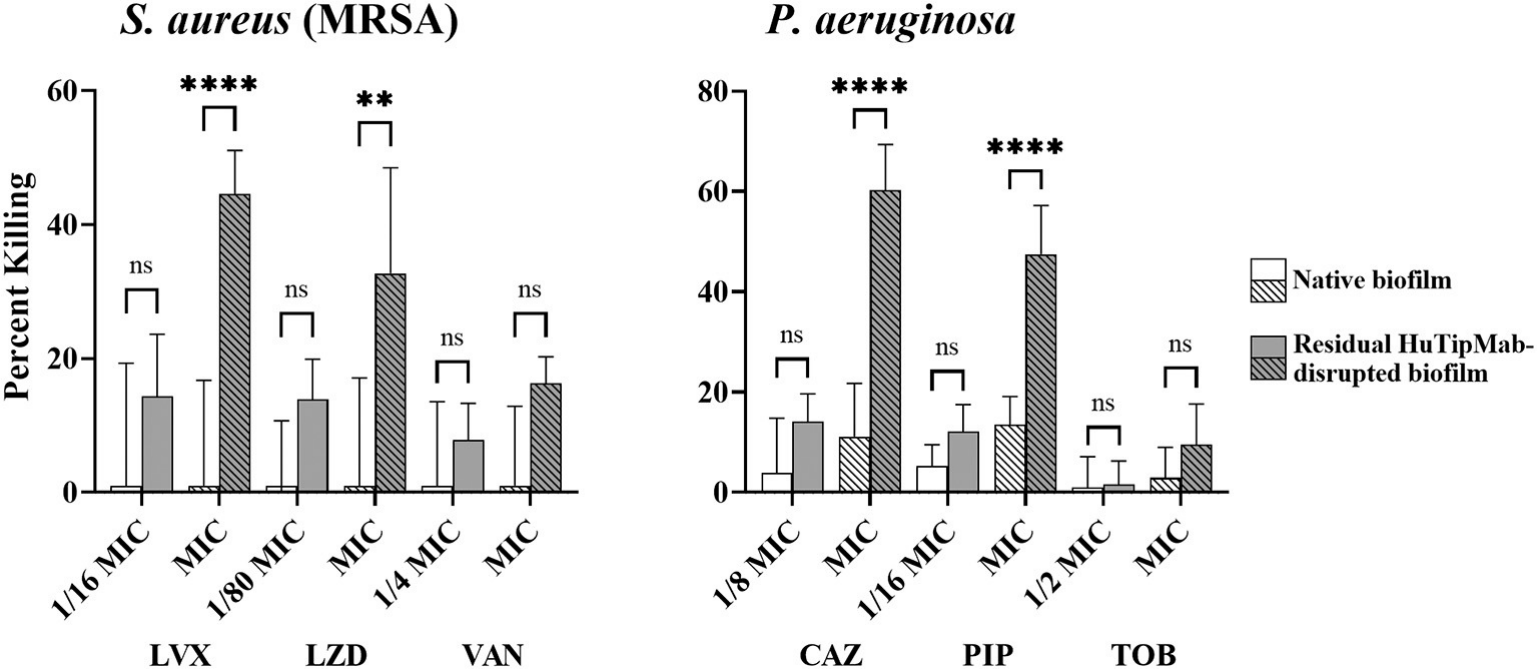
Relative mean percent killing of native biofilms or residual HuTipMab-disrupted biofilms of gram-positive or gram-negative ESKAPEE pathogens. Native biofilms (e.g., incubated with the medium alone) formed by either gram-positive MRSA or gram-negative *Pseudomonas aeruginosa* showed minimal killing when antibiotics were used at a fraction of MIC or full MIC. The killing of native biofilms formed by MRSA was 0% across all tested antibiotics and concentrations by LVX, LZD, and VAN, whereas the killing of native biofilms formed by *P. aeruginosa* by CAZ, PIP, and TOB was 0% at 1/2 the MIC for TOB and ranged from 3 to 13% for CAZ, PIP, and the full MIC of TOB. Residual biofilms of MRSA or *P. aeruginosa* after HuTipMab disruption were killed at a significantly greater extent than native biofilms. At the full MIC of LVX and LZD, the killing of residual MRSA biofilms was 45 and 33%, respectively, significantly greater than their native biofilm counterparts at 0%. Similarly, at the full MIC of CAZ and PIP, residual *P. aeruginosa* biofilms were killed significantly more than their native counterparts (60% vs. 11% for CAZ, 48% vs. 13% for PIP). Antibiotics were used at a fraction or at the full planktonic MIC as indicated on the *x*-axis. Statistically significant differences in percent killing are reported as **P ≤ 0.01 and ****P ≤ 0.001.

Collectively, our data demonstrated that, except for *A. baumannii* NRel when tested with TOB, which showed equal sensitivity to their planktonically grown counterparts, the release of ESKAPEE NRel from biofilm residence by HuTipMab resulted in significantly higher percentages of killing by each of the three selected antibiotics from different classes. These antibiotics were tested at a fraction of the MIC compared to their isogenic planktonically grown counterparts. This outcome suggests that even higher levels of the killing of NRel could be achieved by using these antibiotics at their full MIC. As such, while we used sub-MIC antibiotic concentrations for NRel killing assays to demonstrate any increased sensitivity of NRel compared to their planktonically grown counterparts (as, by definition, use of the full MIC would not allow this determination), we have, in this study, tested the NRel of two representative ESKAPEE pathogens (gram-positive MRSA and gram-negative *P. aeruginosa*) at the full MIC. The killing of MRSA NRel when tested against the full MIC of LVX, LZD, and VAN was 98%−100% (Supplementary Table 3), whereas that of *P. aeruginosa* NRel, when tested against the full MIC of CAZ, PIP, and TOB, was uniformly 100% (Supplementary Table 3). Given the significant disruption of the biofilms formed by each tested ESKAPEE pathogen by a single dose and exposure time with HuTipMab, we investigated whether those bacteria that remained within the disrupted biofilm (only 21%−50% of the biofilm remained depending upon the pathogen; see Supplementary Table 2) might be as susceptible to killing by antibiotics as the NRel. In addition, using MRSA and *P. aeruginosa* as representative ESKAPEE pathogens, we tested both native biofilms and those that remained after HuTipMab-mediated disruption and removal of NRel for the relative percent killing by each of the three chosen antibiotics. While the killing of MRSA within a native biofilm by LVX, LZD, or VAN was 0% when tested at either a fraction or the full MIC, as compared to biofilms incubated with the medium alone, the mean killing of MRSA that remained within the HuTipMab-disrupted biofilm ranged from 8 to 45% (Figure 4, left panel; *P* ≤ 0.001 for LVX and LZD at full MIC). For *P. aeruginosa*, when tested against CAZ or PIP, again at either a fraction of the MIC or the full MIC, the mean killing of native biofilms ranged from 0 to 13%, whereas the fraction for those that remained within the HuTipMab-disrupted biofilm ranged from 10 to 60% (Figure 4, right panel; *P* ≤ 0.001 for CAZ and PIP at full MIC). This isolate of *P. aeruginosa* was highly resistant to TOB; thus, as expected, the killing of bacteria within either native or disrupted biofilms was minimal.

## 4. Discussion

The current standard of care for those with bacterial infections is limited to the use of traditional and, often, broad-spectrum antibiotics. Although clinical laboratory susceptibility testing is relied upon to recommend the most effective antibiotic for individuals with infections, widespread failed clinical efficacy, reaching up to 64%, has been observed nonetheless (Sanchez Garcia, 2009; Macia et al., 2014; Ribeiro da Cunha et al., 2019; WHO, 2019; Rose et al., 2022). Potential detrimental sequelae include recurrence, development of heteroresistance (Band and Weiss, 2019), bacterial persistence, and the concomitant promotion of AMR (Windels et al., 2019). Continuance of ineffective antibiotic treatment can also result in nephrotoxicity, ototoxicity, neurotoxicity, and gut dysbiosis, including clinically challenging *Clostridium difficile* infections (Mohsen et al., 2020). This conundrum arises from the fact that the determined MIC of antibiotics is often not predictive of clinical effectiveness (Lim et al., 2013; Macia et al., 2014; Pammi et al., 2014; Trifilio et al., 2015; Tan et al., 2016; Jorge et al., 2018; Orazi and O'Toole, 2019) because susceptibility testing is performed using bacteria grown in a nutrient-rich medium optimized for planktonic growth. The phenotype of bacteria grown in this manner does not represent their truly relevant physiologic state as this lifestyle is limited to a laboratory and does not occur in a natural environment (Dalhoff, 1985; Olivares et al., 2019; Shi et al., 2019; Kolpen et al., 2022). In a disease state, bacteria are commonly found within a biofilm, where the resident bacteria require a greater than 1,000-fold increase in antibiotic concentration to kill them compared to their planktonic, or free-living, counterparts (Nickel et al., 1985; Ceri et al., 1999; Moskowitz et al., 2004; Hoiby et al., 2010; Hengzhuang et al., 2011, 2012), which is not clinically feasible. Consequently, there has been a concerted effort to identify new antibiotics; however, of the few new antibiotics discovered in the past few decades, none have mitigated AMR or improved clinical outcomes against biofilm-associated diseases (WHO, 2019). Even in the ideal hypothetical scenario where bacteria were unable to develop resistance, any new antibiotic would still demonstrate limited efficacy against the bacteria resident within a biofilm at the infection site due to the inherent protective mechanisms of the recalcitrant, three-dimensional structure.

In this study, we have provided evidence in support of a combinatorial approach where we have envisioned the use of HuTipMab for the controlled disruption of a pathogenic biofilm to release the resident bacteria into the highly vulnerable NRel state. Although HuTipMab cannot itself kill biofilm-resident bacteria, when used at the maximally effective dose, this epitope-targeted monoclonal has been demonstrated to reduce a biofilm to a monolayer of bacteria that has been rapidly and effectively cleared by host immune effectors in three tested pre-clinical models of human disease without the need for antibiotic supplementation (Goodman et al., 2011; Novotny et al., 2016, 2019b, 2020, 2021; Freire et al., 2017). Nonetheless, to minimize risk, we envision a regimen wherein HuTipMab would be delivered along with a traditional antibiotic whose effectiveness has been proven during the period when these high-priority pathogens demonstrate the significantly vulnerable NRel phenotype. The use of this combinatorial approach might lead to the eradication of recalcitrant biofilms and facilitate a more effective clinical outcome. When released from biofilm residence, each of the tested ESKAPEE pathogens was significantly more sensitive to killing by the three tested antibiotics compared to their isogenic planktonically grown counterparts. This observation is noteworthy because biofilm-resident bacteria are traditionally known for their high antibiotic tolerance, whereas planktonically grown bacteria have conventionally been considered the most susceptible bacterial lifestyle. In a single instance, HuTipMab-induced ESKAPEE NRel were found to exhibit comparable sensitivity to killing compared to their planktonically grown counterparts (e.g., *A. baumannii* with TOB); this outcome has significant clinical relevance as it represents a notable improvement over the high antibiotic tolerance typically observed in *A. baumannii* within a biofilm at the disease site. Significantly, we additionally tested both MRSA and *P. aeruginosa* to determine if those bacteria that might have remained within a residual HuTipMab-disrupted biofilm were also significantly more sensitive to killing by antibiotics. This was indeed the case for two of the three antibiotics tested, thereby providing further support for this DNABII-directed biofilm disease eradication strategy.

The molecular mechanism(s) that underlies this transient yet significantly sensitized NRel phenotype, as has been demonstrated for multiple pathogens in this study, are not yet fully known. However, several laboratories have shown that, depending on the exact mechanism of release, this enhanced susceptibility of newly released bacteria can be induced by and/or due to the depletion of pyruvate availability (Goodwine et al., 2019), the decreased ability to respire aerobically (Zemke et al., 2014), or the repressed expression of *phoPQ* by the multidrug transport activator BrlR (Chambers et al., 2017), among others. We demonstrated that the NRel of NTHI, as induced by incubation with an antibody directed against a native DNABII protein (e.g., α-DNABII NTHI NRel), are in the metabolic equivalent of the lag phase (Mokrzan et al., 2020). The relative expression of canonical lag phase genes *deaD, artM*, and *fis* is significantly upregulated in α-DNABII NTHI NRel compared to planktonically grown NTHI. This lag phase parallel has also been observed by others for other bacterial species (Chua et al., 2014). Additionally, compared to their planktonically grown counterparts, α-DNABII NTHI NRel displayed a significantly unique proteomics profile that included decreased expression of proteins involved in cell envelope biogenesis (e.g., Lic2a, LicC, and LicD) (Mokrzan et al., 2020), which contribute to the maintenance of the outer membrane barrier, virulence, and resistance (Pang et al., 2008). The expression of proteins involved in lipid metabolism and essential cofactors was also significantly downregulated, whereas that of the major outer membrane porin of NTHI (Andersen et al., 2003), OMP P2, was significantly greater in α-DNABII NTHI NRel compared to planktonically grown NTHI (Mokrzan et al., 2020); each of which could facilitate greater access of antibiotics into the bacterial cell. Although this differential expression of genes and proteins was assessed at a single time point, providing only a snapshot of α-DNABII NTHI NRel characteristics, *in vitro*, this phenotype becomes apparent within minutes and persists for ~6 h (Mokrzan et al., 2020). Similar vulnerable periods lasting several hours have been observed for other bacterial pathogens newly released from biofilm residence (Marks et al., 2013; Chao et al., 2014; Chua et al., 2014; Chambers et al., 2017). A limitation of our study is that we tested only a single clinical isolate of each ESKAPEE pathogen, limiting the extension of our observations beyond the tested strains. However, in our previous research, we reported the same significant sensitivity to antibiotic-mediated killing NRel phenotype for an additional isolate of three ESKAPEE pathogens: *A. baumannii, S. aureus*, and *P. aeruginosa* (Kurbatfinski et al., 2022).

Given that, unless they are killed, NRel could potentially re-establish a pathogenic biofilm either at the original disease site or elsewhere in the body, we propose a novel therapeutic regimen that involves the simultaneous delivery of HuTipMab and an effective antibiotic. This combinatorial approach is designed to leverage the window of opportunity provided by the highly vulnerable NRel phenotype to improve the clinical outcome for those with recalcitrant disease due to an ESKAPEE pathogen and, perhaps, reduce further contribution to AMR by reducing the antibiotic treatment course and the effective dose of that antibiotic or, ideally, both. The new data presented in this study provide additional support for our ongoing development of this species-agnostic, combinatorial approach.

## Data availability statement

The raw data supporting the conclusions of this article will be made available by the authors, without undue reservation.

## Author contributions

NK and CK performed data curation and visualization. NK and LB oversaw the experiments, analysis, manuscript writing, and data interpretation. LB was responsible for supervision and resources. LB and SG conceptualized and obtained funding for this study. All authors contributed to the article and approved the submitted version.
