# Corrigendum: ESKAPEE pathogens newly released from biofilm residence by a targeted monoclonal are sensitized to killing by traditional antibiotics

**DOI:** 10.3389/fmicb.2024.1382491

**Published:** 2024-02-15

**Authors:** Nikola Kurbatfinski, Cameron N. Kramer, Steven D. Goodman, Lauren O. Bakaletz

**Affiliations:** ^1^Center for Microbial Pathogenesis, Abigail Wexner Research Institute at Nationwide Children's Hospital, Columbus, OH, United States; ^2^Department of Pediatrics, The Ohio State University College of Medicine, Columbus, OH, United States

**Keywords:** AMR, MRSA, humanized monoclonal antibody, DNABII proteins, tip-chimer peptide

In the published article, there was an error in the Funding statement. The funding statement is missing credit to the National Institutes of Health for a grant awarded to the corresponding author. Grant NIH R01DC003915 to LB should be added to the funding statement. The correct Funding statement appears below.

