# Supplementary material for: ESKAPEE pathogens newly released from biofilm residence by a targeted monoclonal are sensitized to killing by traditional antibiotics
Source: Front Microbiol. 2023 Jul 26;14:1202215. doi: 10.3389/fmicb.2023.1202215 (PMC10410267; doi:10.3389/fmicb.2023.1202215)
Supplement: Supplementary file 1 [file Presentation_1.zip › Supplementary Table 1.docx]

Supplementary Material

ESKAPEE Pathogens Newly Released from Biofilm Residence by a Targeted Monoclonal are Sensitized to Killing by Traditional Antibiotics

Nikola Kurbatfinski, Cameron N. Kramer, Steven D. Goodman, Lauren O. Bakaletz*

*** Correspondence:** Corresponding Author: Lauren.Bakaletz@nationwidechildrens.org

# Supplementary Figures and Tables

| **Supplemental Table 1. Organisms used and source** | | |
| --- | --- | --- |
|  |  |  |
| Organism | Strain | Original source of isolates |
|  |  |  |
| *A. baumannii* | 25157 | Burn wound isolate, Clinical Microbiology laboratory at Nationwide Children’s Hospital, Columbus, OH |
|  |  |  |
| *E. coli* | UTI89 | Urinary tract infection (UTI) isolate ([Goodman et al., 2011](#_ENREF_1)) |
|  |  |  |
| *E. faecium* | Com12 ([Palmer et al., 2012](#_ENREF_2)) | Human fecal isolate |
|  |  |  |
| *Enterobacter* sp. |  | UTI isolate, Clinical Microbiology laboratory at Nationwide Children’s Hospital, Columbus, OH |
|  |  |  |
| *K. pneumoniae* | 13883 | UTI isolate, ATCC |
|  |  |  |
| *P. aeruginosa* | 27853 | Blood culture isolate, ATCC |
|  |  |  |
|  | Clinical isolate | Pediatric cystic fibrosis sputum isolate |
|  |  |  |
| *S. aureus* | 29213 | Wound isolate, ATCC |
|  |  |  |
|  | MRSA isolate | Pediatric cystic fibrosis isolate |

**Supplemental Materials Reference List**

Goodman, S.D., Obergfell, K.P., Jurcisek, J.A., Novotny, L.A., Downey, J.S., Ayala, E.A., et al. (2011). Biofilms can be dispersed by focusing the immune system on a common family of bacterial nucleoid-associated proteins. *Mucosal Immunol.* 4, 625-637. doi: 10.1038/mi.2011.27.

Palmer, K.L., Godfrey, P., Griggs, A., Kos, V.N., Zucker, J., Desjardins, C., et al. (2012). Comparative genomics of enterococci: variation in *Enterococcus faecalis*, clade structure in *E. faecium*, and defining characteristics of *E. gallinarum* and *E. casseliflavus*. *mBio* 3, e00318-00311. doi: 10.1128/mBio.00318-11.
