# Supplementary material for: ESKAPEE pathogens newly released from biofilm residence by a targeted monoclonal are sensitized to killing by traditional antibiotics
Source: Front Microbiol. 2023 Jul 26;14:1202215. doi: 10.3389/fmicb.2023.1202215 (PMC10410267; doi:10.3389/fmicb.2023.1202215)
Supplement: Supplementary file 1 [file Presentation_1.zip › Supplementary Table 2.docx]

Supplementary Material

ESKAPEE Pathogens Newly Released from Biofilm Residence by a Targeted Monoclonal are Sensitized to Killing by Traditional Antibiotics

Nikola Kurbatfinski, Cameron N. Kramer, Steven D. Goodman, Lauren O. Bakaletz*

*** Correspondence:** Corresponding Author: Lauren.Bakaletz@nationwidechildrens.org

# Supplementary Figures and Tables

| **Supplemental Table 2. Ability of Tested Antibiotics to Kill NRel of a representative Gram negative and positive ESKAPEE Pathogen When Used at the Full MIC** | | | | |
| --- | --- | --- | --- | --- |
| **ESKAPEE Pathogen** | **Abx** | **Concentration (μg/ml)** | **Percent Killing** |  |
| *S. aureus* (MRSA) | LVX | 0.125 | 100% |  |
|  | LZD | 2 | 98% |  |
|  | VAN | 1 | 100% |  |
| *P. aeruginosa* | CAZ | 2 | 100% |  |
|  | PIP | 2 | 100% |  |
|  | TOB | 2048 | 100% |  |
