# Supplementary material for: ESKAPEE pathogens newly released from biofilm residence by a targeted monoclonal are sensitized to killing by traditional antibiotics
Source: Front Microbiol. 2023 Jul 26;14:1202215. doi: 10.3389/fmicb.2023.1202215 (PMC10410267; doi:10.3389/fmicb.2023.1202215)
Supplement: Supplementary file 1 [file Presentation_1.zip › Supplementary Table 3.docx]

Supplementary Material

ESKAPEE Pathogens Newly Released from Biofilm Residence by a Targeted Monoclonal are Sensitized to Killing by Traditional Antibiotics

Nikola Kurbatfinski, Cameron N. Kramer, Steven D. Goodman, Lauren O. Bakaletz*

*** Correspondence:** Corresponding Author: Lauren.Bakaletz@nationwidechildrens.org

# Supplementary Figures and Tables

| **Supplemental Table 3. Mean Percent Disruption of Biofilms Formed by ESKAPEE Pathogens by Single Tested Dose and Treatment Period with HuTipMab** | |
| --- | --- |
| **ESKAPEE pathogens** | **Mean Percent Disruption ± SEM** |
| *E. faecium* | 72% ± 9% |
| *S. aureus* (MRSA) | 69% ± 0.5% |
| *K. pneumoniae* | 79% ± 2% |
| *A. baumannii* | 64% ± 2% |
| *P. aeruginosa* | 50% ± 9% |
| *Enterobacter* sp. | 72% ± 1% |
| *E. coli* | 72% ± 1% |
